# The Importance of a Pre-treatment Baseline When Screening Patients for Breast-Cancer-Related Lymphedema

**DOI:** 10.1245/s10434-025-17854-0

**Published:** 2025-07-23

**Authors:** Steven L. Chen, Chirag Shah, John Boyages, Louise Koelmeyer, Frank A. Vicini, Sheila H. Ridner

**Affiliations:** 1 OasisMD, San Diego, CA USA; 2 Department of Radiation Oncology, Allegheny Health Network, Pittsburgh, PA USA; 3 Australian Lymphoedema Education, Research and Treatment (ALERT) Centre, Macquarie University, Sydney, Australia; 4 Michigan Healthcare Partners, Farmington Hills, MI USA; 5 Vanderbilt University School of Nursing, Nashville, TN USA

**Keywords:** Breast cancer, Lymphedema, Bioimpedance spectroscopy (BIS), L-Dex, Prospective surveillance, PREVENT

## Abstract

**Background:**

The PREVENT randomized clinical trial showed that bioimpedance spectroscopy (BIS) screening for subclinical breast-cancer-related lymphedema (sBCRL) detection with early intervention is associated with lower progression to chronic breast-cancer-related lymphedema (cBCRL) than tape measurement (TM). This study explores the role of a pre-treatment baseline in detecting sBCRL.

**Methods:**

A total of 1200 patients with breast cancer were randomized for screening using L-Dex (BIS) or relative interlimb volume difference (RILVD) from TM, with 918 patients being monitored for up to 36 months. sBCRL was identified in 209 patients by either an increase from baseline of ≥ 6.5 L-Dex units (*n* = 89) or ≥ 5% and < 10% increase in RILVD (*n* = 120). The pre-treatment and sBCRL trigger L-Dex and RILVD were analyzed using Mann–Whitney, independent *t*-tests, chi-squared, and analysis of variance (ANOVA) tests.

**Results:**

The baseline L-Dex or RILVD was less than zero in 587 (48.9%) and 596 (49.0%) patients, respectively. At sBCRL trigger, 70.8% BIS patients had L-Dex inside the normal range (L-Dex < 10), and 40.4% had L-Dex < 6.5, which could result in misclassification without a pre-treatment baseline. For TM patients, 87.5% had an RILVD inside the normal range (RILVD < 10%), and 51.7% had a RILVD < 5%, which would also cause misclassification without a pre-treatment baseline.

**Conclusions:**

Many patients who triggered for sBCRL would not have been identified without a pre-treatment baseline, potentially missing opportunities for early intervention and increasing the risk of progression. Regardless of the measurement technique used, a recorded baseline is crucial for accurate detection and timely management of sBCRL.

The potential for development of lymphedema is a fear expressed by many patients upon completion of treatment for breast cancer. The onset of lymphedema is characterized by the accumulation of lymph fluid due to a compromised lymphatic system, which can lead to a chronic condition, increasing the risk of infection, cellulitis, and sepsis. This not only impacts a patient’s physical health but also significantly affects the quality of life and emotional well-being of cancer survivors.

Previous studies have shown that the use of a prospective surveillance model of care for the early detection of subclinical breast-cancer-related lymphedema (sBCRL) with early intervention can improve patient outcomes.^1–3^ More recently, the PREVENT randomized controlled trial demonstrated that bioimpedance spectroscopy (BIS)-guided surveillance was associated with lower rates of progression to chronic breast cancer-related lymphedema 7(cBCRL) as compared with tape measure (TM).^4–7^

Best practices for monitoring patients at risk of sBCRL using BIS includes the establishment of a baseline reading prior to treatment, against which the post-treatment measurements can be compared.^8–11^ Regardless of monitoring technique, thresholds for detection are based on tracking post-treatment changes from a baseline measurement. This manuscript reports a secondary analysis of the PREVENT study and examines the impact of determining the pre-treatment baseline in detecting sBCRL when screening using both BIS and TM.

## Methods

The PREVENT trial was a multicenter, international, randomized controlled trial that compared the identification of sBCRL using L-Dex measured by BIS against relative interlimb volume difference (RILVD) measured by circumferential TM in reducing the rates of progression to cBCRL when coupled with early lymphedema intervention up to 3 years post-surgery. The trial patient population consisted of patients over 18 years of age with an initial diagnosis of histologically confirmed invasive breast cancer or ductal carcinoma in situ. Patients with prior breast cancer diagnosis or treatment, or lymphedema were excluded from the study. Other exclusion criteria were linked to measurement method contraindications.

The study was registered at clinicaltrials.gov (NCT02167659), and all trial protocols were approved by the relevant institutional review boards (IRB) for ethics compliance and Scientific Review Committees for scientific integrity. The study was conducted under Good Clinical Practice (GCP) guidelines. Primary and secondary outcome analysis and consort diagrams have been published elsewhere,^7,12–15^ and a summary of the methodology is now provided.

BIS measurements were taken using the L-Dex U400 (ImpediMed Limited, Australia) as detailed elsewhere.^7,12–15^ TM circumferences were measured twice at 10-cm intervals up to 50 cm along the arm. The volume of each arm was calculated using the average of the two measurements in a truncated cone formula, and the average volume was used to determine the RILVD.

Baseline measurements were taken using both measurement techniques prior to cancer treatment, and patients were then randomized to a measurement follow-up group (BIS or TM) during which time only the one monitoring method was used until the patient triggered for sBCRL or the last visit of the study. Monitoring visits were scheduled 3, 6, 12, 18, 24, 30, and 36 months post treatment with optional visits at 15 and 18 months. A patient detected as having sBCRL by BIS or TM at any visit post-cancer treatment triggered early intervention and additional measurement by the alternative monitoring method. Detection of sBCRL was not considered at baseline. In the BIS screening group, sBCRL was defined as an L-Dex score that had increased by 6.5 or higher above the pre-treatment baseline measurement. In the TM screening group, sBCRL was defined as an increase in RILVD greater than or equal to 5% but lower than 10% above the pre-treatment baseline.

Early intervention required patients to wear a 22–32 mmHg compression sleeve and gauntlet for 12 h per day for a total of 4 weeks. At the end of this intervention, patients were again measured by both monitoring methods, and the outcome of the treatment was determined. Patients whose primary measurement method returned to within pre-treatment baseline levels continued to be monitored for the duration of the study. On the other hand, patients whose RILVD rose above 10% higher than their pre-treatment baseline were considered to have progressed to cBCRL and referred directly for complex decongestive physiotherapy (CDP). This could occur at any point in the study, and some patients were referred directly to CDP without undergoing any intervention treatment for sBCRL as their RILVD rose greater than 10% above the pre-treatment baseline between visits. This secondary analysis includes all patients with a pre-treatment baseline measurement. The distribution of pre-treatment baselines for both BIS L-Dex scores and TM RILVD was calculated as well as the absolute L-Dex and RILVD values at the time at which sBCRL was triggered on the basis of the relevant threshold change from baseline. The baseline values were compared across age, BMI, stage of cancer, and cancer treatment as well as sBCRL triggers and cBCRL progression. Comparisons were performed using Mann–Whitney, independent *t*-tests, chi-squared, and analysis of variance (ANOVA) tests.

The primary aim was to assess the value of individualized baseline data in accurately identifying sBCRL and reducing the risk of misclassification when relying solely on population-based reference thresholds. We calculated the distribution of L-Dex and RILVD baseline values and compared them with absolute values at the time of sBCRL trigger.

Published studies have shown higher sensitivity and specificity of single-timepoint BIS measures can be achieved using a lower detection threshold.^16,17^ Therefore, the proportion of patients whose values would fall below conventional diagnostic thresholds (i.e., L-Dex < 6.5 or < 10; RILVD < 5% or < 10%) was determined to quantify the impact of missing baselines on potentially underreporting sBCRL.

To explore potential associations between patient characteristics and baseline variability, we stratified baseline L-Dex and RILVD values by age, body mass index (BMI), race, and cancer stage and assessed whether these demographic or clinical variables influenced baseline distributions and could confound the interpretation of follow-up measurements. All statistical comparisons were performed using Mann–Whitney *U* tests, independent *t*-tests, chi-squared tests, and one-way ANOVA, as appropriate. 

## Results

The study enrolled 1200 patients whose BIS (L-Dex) and TM (RILVD) baselines were recorded. Of these, 918 patients were monitored for sBCRL using either BIS (*n* = 461) or TM (*n* = 457). sBCRL was identified in 209 patients (22.7%, BIS: *n* = 89 (19.3%), TM: *n* = 120 (26.3%), *P* = 0.014)

Of the 209 patients who triggered for sBCRL and underwent intervention, 30 patients (14.4%) progressed to cBCRL (BIS: *n* = 7 (7.9%), TM: *n* = 23 (19.2%), *P* = 0.016). In addition, 39 patients (4.2%) progressed to cBCRL between visits and were referred directly to CDP without a preceding trial of compression alone. A summary of patient demographics and treatment characteristics is presented in Table 1.

**Table 1:** Patient baseline demographics

	Baseline measures
No. of participants	1200
Age at baseline (years)	58 (28-93)
BMI at baseline (kg/m^2^)	29.2 (16–61)
Race	
Asian	107 (8.9%)
Black or African American	93 (7.8%)
White	917 (76.4%)
Multiracial or Other	57 (4.8%)
Missing	26 (2.2%)
Ethnicity	
Non-Hispanic or Latina	1092 (91.0%)
Hispanic or Latina	44 (3.7%)
Missing/do not care to respond	64 (5.4%)
Stage of cancer	
0 (DCIS)	66 (5.5%)
I	634 (52.8%)
II	371 (30.9%)
III	91 (7.6%)
IV	12 (1.0%)
Missing	26 (2.2%)
Type of breast surgery	
Conservative surgery only	850 (70.8%)
Mastectomy and conservative surgery	308 (25.7)
Missing	42 (3.5%)
Axillary surgery	
ALND	241 (20.1%)
SNB only	862 (71.8%)
Other	14 (1.2%)
No node surgery	63 (5.3%)
Missing	20 (1.7%)
Chemotherapy	
None	678 (56.5%)
Without taxane	69 (5.8%)
With taxane	430 (35.8%)
Missing	23 (1.9%)
Radiation therapy	
None	325 (27.1%)
Breast/chest wall	832 (69.3%)
Supraclavicular fossa	137 (11.4%)
Infraclavicular fossa	17 (1.4%)
Internal mammary chain	79 (6.6%)
Axilla level 1	64 (5.3%)
Axilla level 2	34 (2.8%)
Axilla level 3	26 (2.2%)
Missing	26 (2.2%)
Endocrine therapy	
Some	363 (30.3%)

**Fig. 1:**
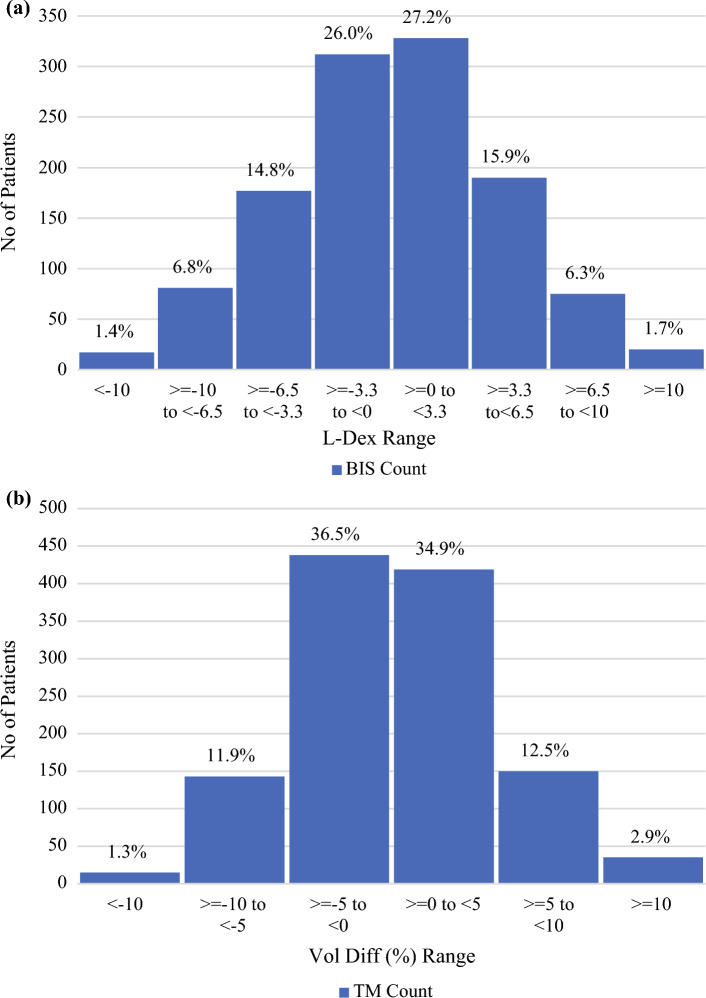
(**a**) Distribution of BIS-based L-Dex scores at baseline for 1200 patients, pre-cancer treatment. (**b**) Distribution of TM-based relative inter-arm volume differences at baseline for 1200 patients, pre-cancer treatment

The distribution of the 1200 baseline L-Dex scores and RILVD are shown in Fig. 1a and b. The baselines for both monitoring methods are normally distributed around a mean of approximately zero (L-Dex: *P* = 0.1187, RILVD: *P* = 0.1154).

The mean L-Dex baseline in Fig. 1a is 0.0 with a standard deviation of 4.8 units. Consequently, 587 patients (48.9%) had a pre-treatment baseline L-Dex score that was negative or below zero, as expected of a normal distribution. The expected reference range for L-Dex scores is between −10 and +10. 17 patients (1.4%) had a pre-treatment baseline L-Dex < −10, and 20 patients (1.7%) had a pre-treatment L-Dex > 10.

The mean RILVD in Fig. 1b is 0.2% with a standard deviation of 4.8%, and 596 patients (49.0%) had a pre-treatment baseline RILVD that was negative or below zero. The reference range for RILVD is generally accepted as between −10% to +10%. In this dataset, 15 patients (1.3%) had a pre-treatment baseline < −10%, and 35 patients (2.9%) had a pre-treatment baseline > 10%, totaling 4.2% of pre-treatment baseline measurements.

**Fig. 2:**
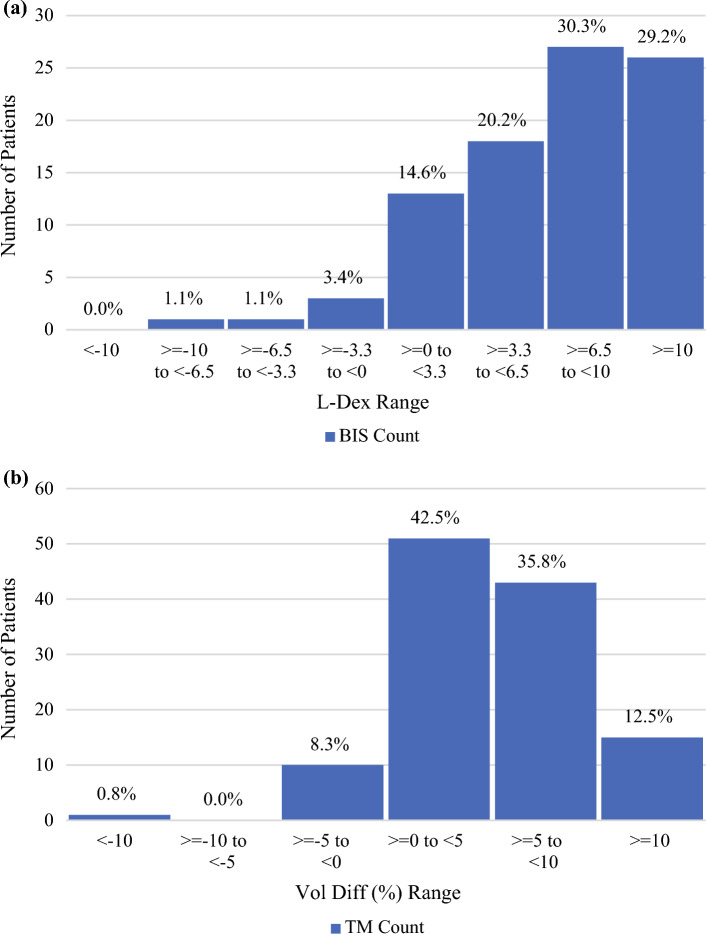
(**a**) Distribution of L-Dex scores at trigger visit for 89 sBCRL patients monitored using BIS. (**b**) Distribution of relative inter-arm volume difference at trigger visit for 120 sBCRL patients monitored using TM

The distribution of the value of the absolute L-Dex score at the time of detection for the 89 patients who triggered for sBCRL via BIS is shown in Fig. 2a. The mean L-Dex score was 4.4 with a standard deviation of 7.6. Only 26 of 89 (29.2%) of patients monitored using BIS had an L-Dex score outside the standard reference range (i.e., L-Dex ≥ 10) at the time of sBCRL trigger. In the absence of a baseline to compare against, at the time of the sBCRL trigger, 63 patients (70.8%) would have been misclassified on the basis of absolute L-Dex scores of < 10 alone and as a result missed out on the opportunity for early intervention.

Using an absolute L-Dex score of 6.5 as the trigger threshold for lymphedema instead of 10 assumes that the patient started with a baseline of zero. Using this threshold of 6.5, 53 of 89 patients (59.6%) had an absolute L-Dex score ≥ 6.5, leaving 36 (40.4%) patients with a putatively normal L-Dex score of < 6.5 who would still be misclassified without the pre-treatment baseline for comparison.

The distribution of the value of RILVD at the time of detection for the 120 patients who triggered for sBCRL via TM is shown in Fig. 2b. The mean RILVD was 4.0% with a standard deviation of 5.1%. For the patients monitored using TM, 15 of 120 (12.5%) patients had a RILVD outside the normal range (RILVD ≥ 10%) at the time of sBCRL trigger, and 58 of 120 patients (48.3%) had a RILVD > 5%, which may be used as a surrogate for detecting sBCRL with only a single-timepoint measure. Had a pre-treatment baseline not been measured, 62 (51.7%, RILVD > 5%) to 105 (87.5%, RILVD ≥ 10%) of TM-monitored patients would also be misclassified. This is summarized in Table 2 for both BIS and TM groups, and while high in both groups, the BIS group had slightly lower misclassification rates. Patients who started at an elevated baseline were not statistically different from the overall group in terms of the number of patients that triggered on the basis of a change in L-Dex or RILVD from baseline as the indicators of sBCRL and cBCRL, or in terms of those who resolved or progressed once treated for their lymphedema.

**Table 2:** Misclassification rate without a pre-treatment baseline

	LDex < 6.5, RILVD < 5% at trigger	LDex < 10, RILVD < 10% at trigger
BIS group (*n* = 89)	36 (40.4%)	63 (70.8%)
TM group (*n* = 120)	62 (51.7%)	105 (87.5%)

Table 3 shows the pre-treatment baseline values for L-Dex and RLIVD, stratified by age, BMI, race, and stage of cancer. One way analysis of variance shows no significant influence of any of the variables for both L-Dex and RILVD baselines. Notably, patients with stage IV cancer showed a trend toward a higher baseline L-Dex (3.9 versus −0.3 to +0.1), although this represented only 1% of the population.

**Table 3:** Baseline measurements stratified by demographics and cancer stage

Stratification	*n*	L-Dex (µ)	RILVD (µ)
BMI group		*P* = 0.095	*P* = 0.836
Underweight	7	0.7	−0.7
Healthy	330	−0.5	0.0
Overweight	417	0.0	0.3
Obese	444	0.4	0.1
Age at diagnosis (years)		*P* = 0.061	*P* = 0.826
< 40	59	−1.1	−0.4
40–50	272	0.1	0.1
50–60	381	−0.3	0.2
60–70	353	0.5	0.3
70+	134	−0.1	0.0
Race		*P* = 0.422	*P* = 0.358
Asian	107	−0.2	−0.2
Black	93	−0.3	0.9
White	916	0.1	0.2
Multiracial or other	57	−1.1	−0.5
Stage of cancer		*P* = 0.068	*P* = 0.415
0	66	−0.3^a^	1.0
I	633	−0.1^b^	0.1
II	371	−0.1^c^	0.0
III	91	0.1	0.5
IV	12	3.9^a,b,c^	1.6

Superscripts denote significant differences

## Discussion

This analysis demonstrates the importance of obtaining a pre-treatment baseline for comparison to maximize sensitivity and specificity for detecting sBCRL regardless of technique. As expected, nearly half of patients start with a negative L-Dex reading or negative RILVD. For BIS, this is expected in that the original reference ranges for healthy patients centered on an L-Dex of zero. Thus, when looking for a change from baseline, an absolute L-Dex score within the reference ranges may be abnormal for any specific patient and indicative of lymphedema. Similarly, while baseline L-Dex scores outside of the reference ranges are uncommon (~3% of patients), they remain possible, either from pre-existing issues or just through normal variation. This may also represent a physiologic response to cancer, especially lymphatic obstruction or metastasis as noted by the higher average BIS baseline for stage IV patients, although this was a small proportion of the population. Similarly, increased baseline lymphedema rates have been observed at diagnosis in other stage IV cancers such as head and neck cancer.^18^

Failure to obtain a baseline RILVD calculation based on TM exhibits similar issues for those followed in this manner. Again, as expected, nearly half of patients had an initial baseline RILVD below zero. Utilizing a 10% difference instead of a 10% change, 87.5% of lymphedema diagnoses would have been missed, and lowering this threshold to 5%, which would assume a patient had a 5% change from a presumed baseline of 0% RILVD, would still have missed 51.7% of patients with a trigger using TM.

**Fig. 3:**
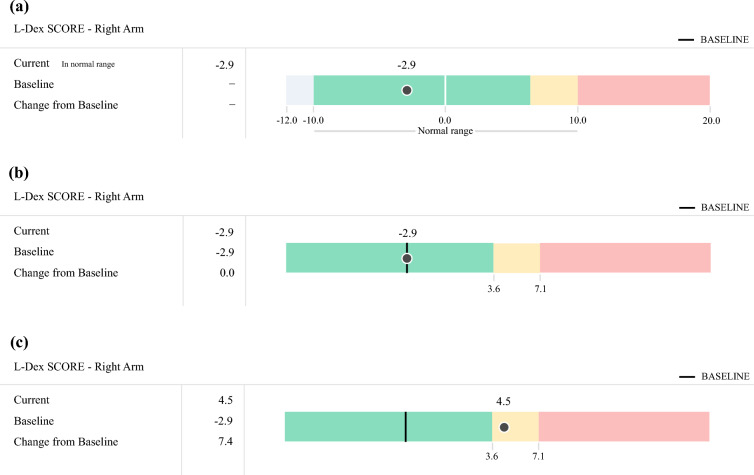
(**a**) No baseline set—thresholds based on population reference ranges. (**b**) Baseline set—thresholds individualized to the patient. (**c**) sBCRL trigger—individual patient threshold met, population threshold not met

Failure to obtain a baseline BIS or RILVD reading may lead these patients to miss an opportunity to be treated early owing to assuming that a measurement is normal for a patient when it is not or potentially overtreating for presumed lymphedema based on an L-Dex score or RILVD that is still normal for that patient given their baseline. As an illustration, Fig. 3 demonstrates an example of the use of L-Dex scores in identifying sBCRL with and without a pre-treatment baseline. Figure 3a shows the L-Dex score measured pre-treatment compared with the detection thresholds based on a matched healthy population that are determined around the mean and the standard deviation from the mean. In this case, the baseline measurement is −2.9 and the detection threshold is 6.5 (yellow) for sBCRL and 10 to be considered outside the healthy range. Figure 3b shows the same measurement, but with the baseline set and the reference ranges individualized to the specific patient. Now the detection thresholds are based on comparison with the baseline and shift to be centered around the patient’s pre-treatment state. Because this patient’s baseline is negative, but still considered normal, to trigger for sBCRL the L-Dex score only needs to reach 3.6 (6.5-unit change) to trigger for sBCRL, as shown in Fig. 3c. If the patient’s baseline was unknown, they would need to experience a much larger post-treatment change from −2.9 to 6.5 (9.4-unit change) before triggering for sBCRL. Thus, patients without a baseline measure but who may naturally have a negative baseline (50% of the population for both BIS and TM) would be delayed in triggering for sBCRL and risk progression to cBCRL when comparing with population reference ranges. This is the first study to clearly demonstrate the importance of baseline measurements in detecting sBCRL at its earliest point by showing the high rate of undetected sBCRL (51.7% to 87.5% depending on measurement method and detection threshold used).

Conversely, for the small subset of patients who had baselines higher than the traditional cutoffs used for patients without a baseline, the failure to obtain a baseline commits many of those patients to starting treatment, albeit a relatively benign one of a short-term compression garment. However, given that they may never resolve to a “normal” L-Dex or RILVD, this may cause patients to persist in treatment even without actual lymphedema.

What should be done in cases where patients do not obtain a pre-treatment baseline? In such cases, a stable, asymptomatic post-treatment measurement may still be helpful and serve as a potential neo-baseline reading with careful consideration to improve the sensitivity of detecting sBCRL onset as early as possible, especially in situations where this neo-baseline reading is below zero. Prospective surveillance consisting of longitudinal measurements should be conducted after this measurement is obtained, and shortening the interval between measurements may be appropriate.

When disease detection thresholds are not based on comparison with an individual’s known healthy and/or stable state, such as a pre-treatment baseline in the case of sBCRL, measurements are instead compared with a matched healthy population dataset and thresholds are based on exceeding a number of standard deviations away from the mean. This can lead to missing triggers for sBCRL as described above for both the BIS and TM monitoring groups, as despite experiencing significant post-treatment changes, the absolute value does not exceed the threshold of detection. Thus, careful attention to patient’s symptomatology in these cases as a supplement to BIS or TM monitoring is essential with a low threshold for considering the use of compression even when BIS or TM may still be within the reference range for the general population. Likewise, for patients without a baseline but a trend toward increasing L-Dex or RILVD, additional measurements in a shorter timeframe may be beneficial.

## Conclusions

Approximately 50% of patients have an established pre-treatment baseline (measured using either BIS or TM) that is less than zero, as expected in a normally distributed variable. Many patients who trigger for sBCRL when monitored from a pre-treatment baseline do not have absolute measurement values exceeding population-based reference ranges centered on zero, the threshold typically used when a baseline was not recorded. These results demonstrate that, regardless of measurement technique, many patients who trigger for sBCRL would not have been detected without a recorded baseline to compare against.

Without this pre-treatment baseline, up to 71% of patients monitored using BIS and up to 88% of patients monitored using TM would not be identified as having sBCRL as early, potentially delaying diagnosis until further progression of the disease increases their measurement outside the expected range. This may result in missing an opportunity for early intervention and risks progression to irreversible cBCRL. This reinforces the recommendation for consideration of baseline lymphedema screening in the NCCN Clinical Practice Guidelines in Oncology and using BIS when it is available.^19,20^

Given the slightly lower misclassification rates using BIS and the reproducibility concerns for TM use, starting BIS monitoring may provide additional information beyond TM, although both may provide benefit since each measures different physiological parameters (impedance versus volume). Ultimately, establishing a pre-treatment or known healthy baseline is vital in detecting sBCRL at the earliest opportunity for ensuring access to early intervention.
